# Predictive role of lymphoscintigraphy undergoing lymphovenous anastomosis in patients with lower extremity lymphedema: a preliminary study

**DOI:** 10.1186/s12880-021-00713-1

**Published:** 2021-12-08

**Authors:** Hye Ryeong Kwon, Ji Hye Hwang, Goo-Hyun Mun, Seung Hyup Hyun, Seung Hwan Moon, Kyung-Han Lee, Joon Young Choi

**Affiliations:** 1 Department of Nuclear Medicine, Samsung Medical Center, Sungkyunkwan University School of Medicine, 81 Irwon-ro, Gangnam-gu, Seoul, 06351 Korea; 2 Department of Physical and Rehabilitation Medicine, Samsung Medical Center, Sungkyunkwan University School of Medicine, Seoul, Korea; 3 Department of Plastic and Reconstructive Surgery, Samsung Medical Center, Sungkyunkwan University School of Medicine, Seoul, Korea

**Keywords:** Lymphedema, Lymphoscintigraphy, Lymphovenous anastomosis, Treatment response

## Abstract

**Background:**

We investigated whether preoperative lymphoscintigraphy could predict the treatment response of unilateral lymphovenous anastomosis (LVA) in patients with lower extremity lymphedema.

**Materials and methods:**

A total of 17 patients undergoing lymphoscintigraphy subsequent to LVA was included. As qualitative lymphoscintigraphic indicators, ilioinguinal lymph node uptake, main lymphatic vessel, collateral vessel, and four types of dermal backflow patterns (absent; distal only; proximal only; whole lower limb) were evaluated. Lymph node uptake ratio, extremity uptake ratio, and injection site clearance ratio were obtained as quantitative lymphoscintigraphic indicators at 1 and 2-h after injection. To evaluate therapy response, the volume difference ratio of the whole lower limb at 3 months (early response) and 1 year (late response) was measured. Volume difference ratios (continuous variable and binary variable with a cut-off value of zero) were compared according to the lymphoscintigraphic variables.

**Results:**

The group with whole lower limb dermal backflow had a greater volume change than the other groups (*p* = 0.047). The group with dermal backflow in the whole lower limb OR only in the distal part had a higher rate of volume reduction than the group with dermal backflow only in the proximal part OR absent (*p* = 0.050). The 2-h extremity uptake ratio was the only indicator that positively correlated with early and late volume difference ratio (*p* = 0.016, *p* = 0.001). The rate of volume decrease at 1 year was high in patients with high 2-h extremity uptake ratio (*p* = 0.027). As the amount of dermal backflow increases, the postoperative therapeutic effect increases (*p* = 0.040).

**Conclusions:**

Preoperative lymphoscintigraphy is useful to predict both early and late therapy response in patients with lower extremity lymphedema undergoing LVA. Both dermal backflow pattern and extremity uptake ratio may be predictive lymphoscintigraphic indicators.

## Background

Lymphedema is defined as interstitial edema and protein accumulation due to defects in lymphatic drainage [1]. Chronic edema causes decreased physical activity, repeated infections, and skin changes, resulting in poor quality of life [2, 3]. Therefore, early diagnosis and treatment for lymphedema are important. The number of female patients with lymphedema is increasing because of the high incidence of secondary lymphedema in breast cancer or gynecological cancer patients receiving lymph node dissection or radiotherapy [4, 5].

In the early stage of lymphedema, complex decongestive therapy and exercise are used as conservative therapy. Lymphovenous anastomosis (LVA) or lymph node transfer can be implemented if conservative therapy is ineffective or in cases with a higher stage of lymphedema [6]. LVA is a microsurgery that creates a bypass or shunt between the lymphatic channel and the blood system. End-to-end anastomosis is performed between the lymphatic channel and the venule, which are submillimeters in diameter. Since the introduction of LVA in the 1960s, surgical techniques have developed greatly [7–10]. LVA is a promising treatment to improve the repeated infections [11] and skin changes [12] as well as volume changes in lymphedema.Because LVA is an invasive procedure, identifying the specific patients who might benefit from this treatment is critical.

Lymphoscintigraphy is an effective imaging modality that can be easily performed on a patient. Preoperative lymphoscintigraphy is widely used for differential diagnosis of lymphedema and for determining the extent of the disease [13]. Lymphoscintigraphy is useful in the detection of lymphatic occlusion sites and evaluation of the functional severity of lymph edema at the diagnostic stage [1, 14, 15]. It also is useful for evaluating and predicting treatment response. Research on predicting prognosis of physical therapy including complex decongestive therapy has been mainly performed [16–18]. However, few studies have examined the prediction of treatment response to LVA [19–21].

The purpose of this study was to investigate whether baseline lymphoscintigraphy was useful to predict treatment response and prognosis in patients with lower extremity lymphedema who underwent LVA. In addition, it was evaluated which qualitative or quantitative lymphoscintigraphic indicators best predict therapy response and prognosis.

## Materials and methods

### Patients

This study included an initial 28 consecutive patients with lower extremity lymphedema who underwent unilateral lymphedema surgery at our institution between September 2009 and April 2020. Among them, 3 patients without preoperative lymphoscintigraphy and 1 patient without a preoperative volume evaluation record were excluded. One patient receiving only liposuction but not LVA was excluded, and 3 patients with bilateral lymphedema were excluded. One patient with follow-up loss after surgery and 2 patients with other venous or rheumatologic disease were excluded. Finally, 17 patients with lower extremity edema were enrolled. For all patients, LVA surgery was decided by a Plastic Surgeon (G-H Mun) according to preoperative indocyanine green (ICG) lymphangiography. Lymphatic function was determined by the drainage line and flow rate of dye in ICG lymphangiography, and LVA was performed if functional lymphatics were found on the examination [22]. As postoperative care, all patients used elastic compression stocking of class III degree (30–40 mmHg) and bandages were applied to the distal end. This study was approved by the Institutional Review Board of Samsung Medical Center on 24 June 2021 (IRB File No. SMC 2021-06-148), and informed consent was waived due to the retrospective design.

### Lymphoscintigraphic imaging acquisition and analysis

A total of 148 MBq of ^99m^Tc-tin colloid or ^99m^Tc-phytate was injected into the first and second web spaces between the toes of both feet of the patient. Anterior and posterior images of both lower extremities were obtained immediately after the injection (at 0 min) and, at 1 h and at 2 h using a dual-headed gamma camera (e-cam, Siemens Healthineers, Erlangen, Germany). Patients were encouraged to walk for 15 min after immediate post-injection imaging to promote lymphatic drainage. Quantitative indicators were obtained by setting regions of interest (ROIs) on both lower extremities in the 1 h and 2 h delayed images. Lymph node (LN) uptake (%), extremity uptake (%), and injection site clearance (%) were calculated as follows.

$${\text{LN}}\,{\text{uptake}}\,(\% ) = 100 \times ({\text{ilioinguinal}}\,{\text{LN}}\,{\text{site}}\,{\text{count}}\,{\text{at}}\,1\,{\text{h}}\,{\text{or}}\,2\,{\text{h}})/({\text{injection}}\,{\text{site}}\,{\text{count}}\,{\text{at}}\,0\,\min )$$

$${\text{Extremity uptake }}\left( \% \right) = 100 \times ({\text{extremity count at }}1\,{\text{h or }}2\,{\text{h}})/({\text{injection site count at }}0\,{\text{min}})$$

$${\text{Injection site clearance }}\left( \% \right) = 100 \times ({\text{injection site count at}}\,0\,{\text{min}} - {\text{injection site count at }}1\,{\text{h or }}2\,{\text{h}})/({\text{injection site count at }}0\,{\text{min}})$$

Visual analysis regarding the presence of ilioinguinal LN uptake, visualization of the main lymphatic vessel, visualization of the collateral vessel, and presence of dermal backflow was performed by two nuclear medicine physicians (JY Choi with over 20 years of experience in Nuclear Medicine field & HR Kwon with 5 years of experience) who were unaware of clinical information. The intraclass coefficient for the first reading of both physicians was above 0.9. After that, consensus on the final interpretation was made. Regarding the radiopharmaceutical used, there was no significant difference in diagnostic performance between tin colloid and phytate [23].

### Clinical and imaging variables

Age, sex, clinical stage, etiology (cause) of lymphedema (either primary or secondary), preoperative volume evaluation of the whole lower limb, and postoperative volume evaluation of the whole lower limb (at 3 months and 1 year after surgery) were obtained through review of electronic medical records. Primary lymphedema was diagnosed in patients without conditions causing secondary lymphedema (malignancy, post-infectious/post-therapeutic obstruction, lymphovascular disease, obesity, etc.) and with the appearances of primary lymphedema (spontaneous puffy swelling and then indurated and fibrosed) [24]. Secondary lymphedema patients were diagnosed after gynecological cancer operation (10 cases of cervical cancer, 1 case of endometrial cancer, and 1 case of ovarian cancer). These diagnoses were made by JH Hwang, a Rehabilitation Medicine Physician with more than 10 years of experience in lymphedema treatment. The etiology is important because the onset time and disease duration can differ according to the cause of lymphedema, which may affect the surgical outcome.

The volumes of lower limbs were measured using an electronic volumeter (Perometer; Pero-system, Wuppertal, Germany). When the patient puts his or her lower limb on the plate, the volumetry sensor scans and passes over it, and the 3D volume is automatically calculated. The reproducibility of measurements by volumeter is considered to be reliable [25]. This clinical evaluation was done by technicians under the supervision of Rehabilitation Medicine Physician (JH Hwang). The patients visited the outpatient department of rehabilitation medicine every 3 months after surgery and lower limb volumes were measured at each visit. The volume difference ratio ((preoperative volume minus postoperative volume)/preoperative volume) was used as a treatment response evaluation tool. Volume change after 3 months was regarded as early response, and change after 1 year was indicated as late response. Volume difference ratio was analyzed not only as a continuous variable, but also as a dichotomous treatment response with a cut-off value of zero. A volume difference ratio below 0 was considered a volume increase, and a volume difference ratio above 0 was considered a volume decrease. In addition, history of inflammation before and after surgery was confirmed because of its impact on prognosis [26]. The build-up of fluid in tissues makes lymphedema patients more vulnerable to infection. Heat/fever and redness were mainly shown as clinical manifestations in the patients with cellulitis/infection of the lymphedema site.

**Fig. 1:**
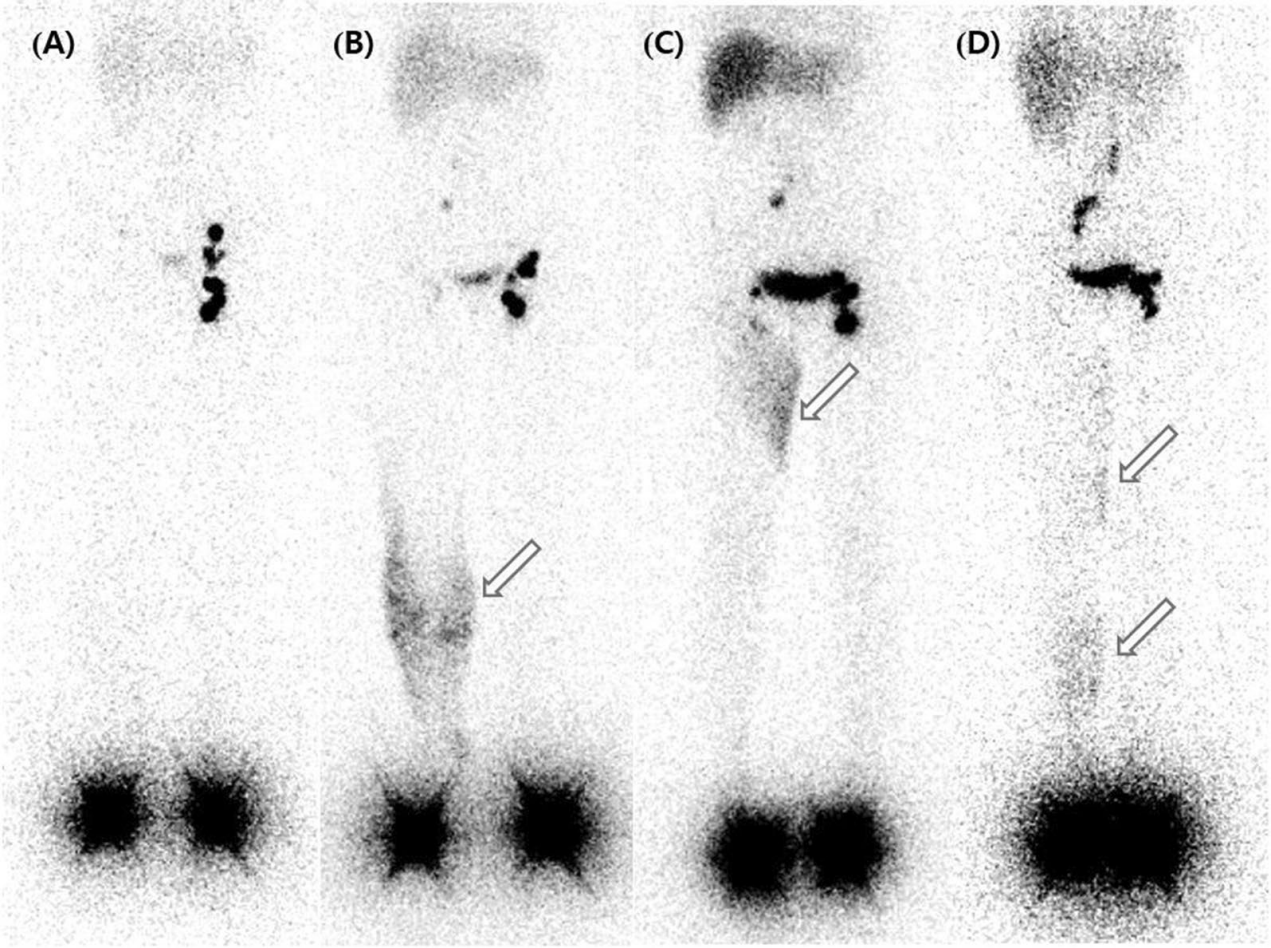
Representative lymphoscintigraphic images according to dermal backflow pattern in the right lower extremity. **A** ‘Absent’ dermal backflow in a 54-year-old female patient. **B** ‘Distal only’ pattern of dermal backflow in a 44-year-old female patient. **C** ‘Proximal only’ pattern of dermal backflow in a 52-year-old female patient. **D** ‘Whole lower limb (both proximal and distal)’ pattern of dermal backflow in a 30-year-old female patient

Imaging indicators were categorized as qualitative and quantitative. As qualitative indicators, ilioinguinal LN uptake, main lymphatic vessel, collateral vessel, and dermal backflow were evaluated by a binary method (absent/present). Dermal backflow was additionally classified into the ‘Distal only’ pattern, ‘Proximal only’ pattern, and ‘Whole lower limb (proximal & distal)’ pattern according to site (Fig. 1). As quantitative indicators, the ratio of edematous limb/healthy limb of each lymphoscintigraphic parameter was used for consideration of individual variance of each absolute value.

### Statistical analysis

Because the patient group was a small cohort and did not satisfy normality, all analyses were conducted in a non-parametric manner. Mann–Whitney test was used to evaluate the relationships between qualitative indicators and volume difference ratio. Fisher's extract test was used to evaluate the relationships between qualitative indicators and dichotomous response. Spearman’s rank correlation was used to evaluate the relationships between quantitative indicators and volume difference ratio. Mann–Whitney test was used to evaluate the relationships between quantitative indicators and dichotomous response. The pattern of dermal backflow was evaluated based on three patterns: absent; distal only OR proximal only; whole lower limb. The Jonckheere Terpstra test was performed to determine the trend in volume difference ratio among the 3 groups, and linear by linear association was performed to determine the trend in dichotomous response among the 3 groups. IBM SPSS Statistics software (version 27.0) was used for analyses, and a *p* value less than 0.05 was considered statistically significant.

## Results

### Patients’ clinical and lymphoscintigraphic characteristics

The clinical and lymphoscintigraphic characteristics of the patients included in this study are listed in Table 1. The mean patient age was 42 ± 11 years and the majority of patients were women (16 female, 1 male). The clinical stage was 2 or 3. Primary lymphedema was observed in 5 and secondary lymphedema was observed in 12. Two or more LVAs were performed on 15 patients. Calf and ankle were the main LVA sites (calf, n = 22; ankle, n = 19; thigh, n = 1).

**Table 1:** Patients’ clinical and lymphoscintigraphic characteristics

Variables	Value or no. of patients
*Age (years)*	
Mean ± SD	42.06 ± 11.01
*Sex*	
Female	16
Male	1
*Lymphedema site (affected limb)*	
Right	8
Left	9
*Clinical stage*	
2	10
3	7
*Etiology*	
Primary	5
Secondary	12
*Inguinal lymph node uptake*	
Absent	7
Present	10
*Main lymphatic vessel*	
Absent	13
Present	4
*Collateral vessel*	
Absent	15
Present	2
*Dermal backflow*	
Absent	5
Present	12
*1 h LN uptake ratio*	
Mean ± SD	0.39 ± 0.53
*1 h Extremity uptake ratio*	
Mean ± SD	1.46 ± 0.50
*1 h Injection site clearance ratio*	
Mean ± SD	1.04 ± 0.26
*2 h LN uptake ratio*	
Mean ± SD	0.45 ± 0.61
*2 h Extremity uptake ratio*	
Mean ± SD	1.42 ± 0.47
*2 h Injection site clearance ratio*	
Mean ± SD	0.97 ± 0.13

### Relationships between clinical findings, volume change, and therapy response

Age, clinical stage, and etiology were not significant factors related to volume change or therapy response. There was also no statistical significance in the relationship between inflammation history and volume change or, inflammation history and dichotomous therapy response. There were no significant differences in the volume change and therapy response according to the number of LVAs (≤ 2 vs. > 2).

### Relationships between lymphoscintigraphic findings and volume change

**Fig. 2:**
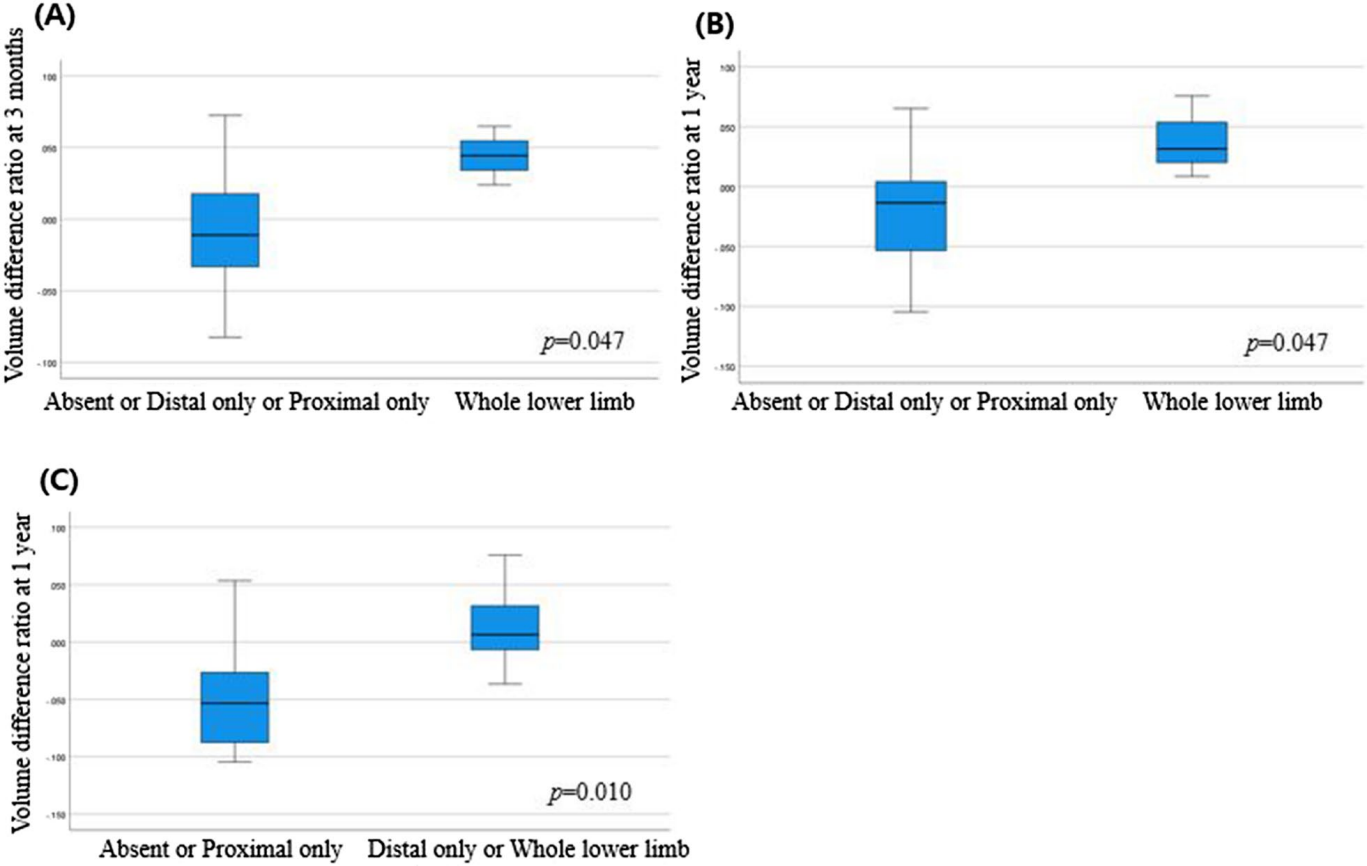
Differences in extremity volume changes according to dermal backflow pattern on preoperative lymphoscintigraphy. **A**, **B** The group with dermal backflow in the whole lower limb had more early and late reduction in extremity volumes after surgery than did other groups. **C** The group with dermal backflow in the whole lower limb or only in the distal part had more late reduction in extremity volumes after surgery than did the group with dermal backflow only in the proximal part or absent

Table 2 and Fig. 2 present the relationships between qualitative indicators and volume difference ratio (at 3 months and at 1 year). The presence/absence of LN uptake, main lymphatic vessel, and collateral vessel were not significantly associated with the volume difference ratio. Dermal backflow patterns were examined in various combinations: pattern 1) absent vs. present (distal only, proximal only, whole lower limb); pattern 2) absent or distal only vs. proximal only or whole lower limb; pattern 3) absent or proximal only vs. distal only or whole lower limb; and pattern 4) absent or distal only or proximal only vs. whole lower limb. Only pattern 4 was significantly associated with the early volume difference ratio (at 3 months). The group with both proximal and distal dermal backflow had a greater postoperative volume change than did the rest of the groups (*p* = 0.047). In the analysis of late volume change, patterns 3 and 4 showed statistically significant associations with volume difference ratio at 1 year. The group with dermal backflow in the whole lower limb or only in the distal part had a greater postoperative volume change than did the group with dermal backflow only in the proximal part or absent (*p* = 0.010). In addition, the group with whole lower limb dermal backflow had a greater volume change after surgery than did the other groups (*p* = 0.047).

**Table 2:** Relationships between lymphoscintigraphic findings and volume change

Qualitative lymphoscintigraphic indicators		Volume difference ratio at 3 months			Volume difference ratio at 1 year		
		N	Mean rank	*p*-value	N	Mean rank	*p*-value
Inguinal LN uptake	Absent	7	8.71	N-S	7	9.71	N-S
	Present	10	9.20		10	8.50	
Main lymphatic vessel	Absent	13	10.23	N-S	13	9.46	N-S
	Present	4	5.00		4	7.50	
Collateral vessel	Absent	15	9.13	N-S	15	9.60	N-S
	Present	2	8.00		2	4.50	
Dermal backflow (Pattern 1)	Absent	5	6.80	N-S	5	5.60	N-S
	Present	12	9.92		12	10.42	
Dermal backflow (Pattern 2)	Absent OR distal only	12	8.08	N-S	12	8.42	N-S
	Proximal only OR whole lower limb	5	11.20		5	10.40	
Dermal backflow (Pattern 3)	Absent OR proximal only	7	6.71	N-S	7	5.29	0.010*
	Distal only OR whole lower limb	10	10.60		10	11.60	
Dermal backflow (Pattern 4)	Absent OR distal only OR proximal only	14	7.86	0.047*	14	7.86	0.047*
	Whole lower limb	3	14.33		3	14.33	

Quantitative lymphoscintigraphic indicators	Volume difference ratio at 3 months		Volume difference ratio at 1 year	
	Correlation coefficient (Spearman’s rho)	*p* value	Correlation coefficient (Spearman’s rho)	*p*-value
1 h LN uptake ratio	− 0.007	N-S	0.020	N-S
1 h Extremity uptake ratio	0.431	N-S	0.456	N-S
1 h Injection site clearance ratio	− 0.336	N-S	-0.130	N-S
2 h LN uptake ratio	0.007	N-S	0.044	N-S
2 h Extremity uptake ratio	0.574	0.016*	0.723	0.001*
2 h Injection site clearance ratio	− 0.034	N-S	0.066	N-S

*Statistically significant; N-S, not statistically significant

**Fig. 3:**
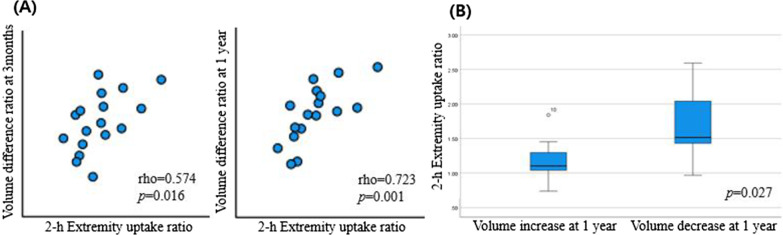
Changes in extremity volumes according to 2-h EUR on preoperative lymphoscintigraphy. **A** The 2-h EUR shows a positive correlation with the early and late volume difference ratio. **B** The group with higher 2-h EUR had a higher rate of volume reduction at 1 year after surgery

Table 2 and Fig. 3 present the relationships between quantitative indicators (edematous limb/healthy limb ratio) and volume difference ratio. Extremity uptake ratio at 2 h (2-h EUR) was significantly associated with volume difference ratio. A higher 2-h EUR was associated with greater volume reduction at 3 months (rho = 0.574, *p* = 0.016). This indicator was significant in relation to late volume change and showed a stronger positive correlation than did the early change (rho = 0.723, *p* = 0.001).

**Fig. 4:**
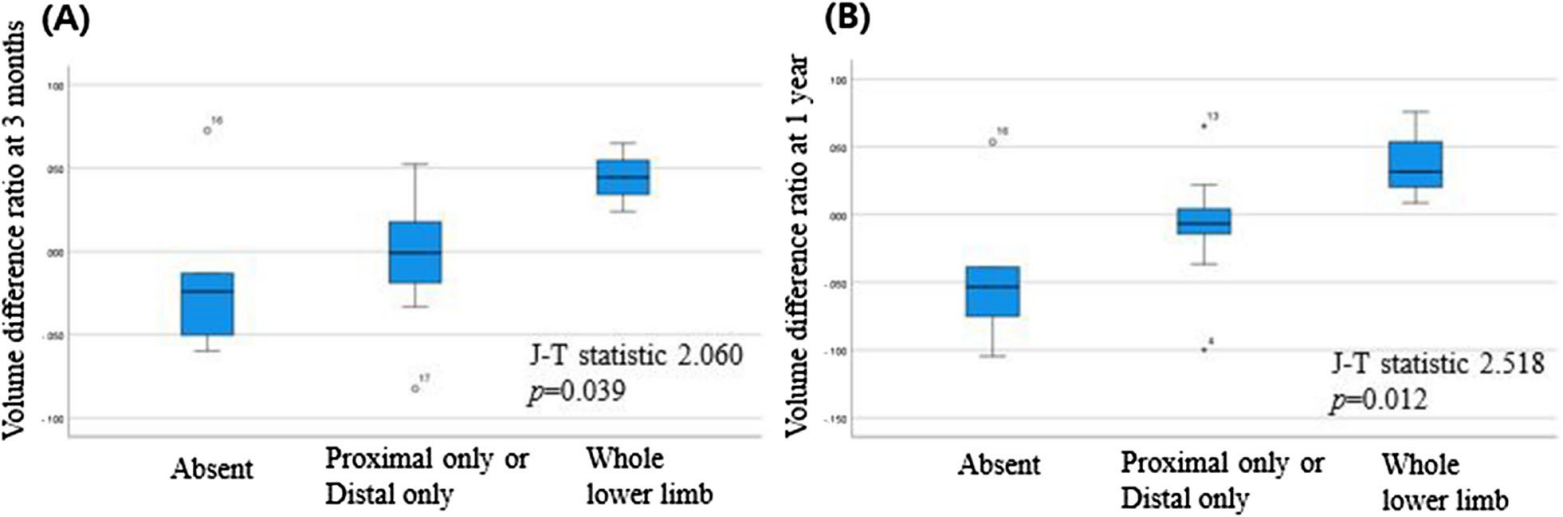
Changes in extremity volumes according to dermal backflow pattern. **A**, **B** The early and late postoperative volume reduction was greater in the group with absent dermal backflow compared with the group with whole lower limb dermal backflow. Standardized J-T statistic is displayed

Trend analysis according to 3 dermal backflow patterns was evaluated and showed results in the order of absent < distal only OR proximal only < whole lower limb (proximal and distal). There was a statistical significance in relation to both early and late volume difference ratio (Fig. 4). The postoperative volume reduction was greater in the group with absent dermal backflow compared with the group with whole lower limb dermal backflow (at 3 months, standardized J-T statistic 2.060, *p* = 0.039; at 1 year, standardized J-T statistic 2.518, *p* = 0.012). Overall, the lymphoscintigraphic indicators predicting early and late volume change were similar.

### Relationships between lymphoscintigraphic findings and therapy response

Table 3 summarizes the relationships between qualitative indicators and dichotomous response (at 3 months and at 1 year). Pattern 3 of dermal backflow was marginally significant. The proportion of volume reduction was higher in the group with dermal backflow in the whole lower limb or only in the distal part compared with the group with dermal backflow only in the proximal part or without dermal backflow (*p* = 0.050).

**Table 3:** Relationships between lymphoscintigraphic findings and therapy response

Qualitative lymphoscintigraphic indicators		Dichotomous early response (at 3 months)			Dichotomous late response (at 1 year)		
		Volume increase^†^	Volume decrease^§^	*p*-value	Volume increase	Volume decrease	*p*-value
Inguinal LN uptake	Absent	4 (57.1%)	3 (42.9%)	N-S	4 (57.1%)	3 (42.9%)	N-S
	Present	5 (50.0%)	5 (50.0%)		5 (50.0%)	5 (50.0%)	
Main lymphatic vessel	Absent	5 (38.5%)	8 (61.5%)	N-S	6 (46.2%)	7 (53.8%)	N-S
	Present	4 (100.0%)	0 (0.0%)		3 (75.0%)	1 (25.0%)	
Collateral vessel	Absent	8 (53.3%)	7 (46.7%)	N-S	7 (46.7%)	8 (53.3%)	N-S
	Present	1 (50.0%)	1 (50.0%)		2 (100.0%)	0 (0.0%)	
Dermal backflow (Pattern 1)	Absent	4 (80.0%)	1 (20.0%)	N-S	4 (80.0%)	1 (20.0%)	N-S
	Present	5 (41.7%)	7 (58.3%)		5 (41.7%)	7 (58.3%)	
Dermal backflow (Pattern 2)	Absent OR distal only	7 (58.3%)	5 (41.7%)	N-S	7 (58.3%)	5 (41.7%)	N-S
	Proximal only OR whole lower limb	2 (40.0%)	3 (60.0%)		2 (40.0%)	3 (60.0%)	
Dermal backflow (Pattern 3)	Absent OR proximal only	6 (85.7%)	1 (14.3%)	0.050	6 (85.7%)	1 (14.3%)	0.050
	Distal only OR whole lower limb	3 (30.0%)	7 (70.0%)		3 (30.0%)	7 (70.0%)	
Dermal backflow (Pattern 4)	Absent OR distal only OR proximal only	9 (64.3%)	5 (35.7%)	N-S	9 (64.3%)	5 (35.7%)	N-S
	Whole lower limb	0 (0.0%)	3 (100.0%)		0 (0.0%)	3 (100.0%)	

Quantitative lymphoscintigraphic indicators		Early response (at 3 months)			Late response (at 1 year)		
		N	Mean rank	*p*-value	N	Mean rank	*p*-value
1 h LN uptake ratio	Volume increase^†^	9	8.22	N-S	9	7.78	N-S
	Volume decrease^§^	8	9.88		8	10.38	
1 h Extremity uptake ratio	Volume increase	9	7.56	N-S	9	7.44	N-S
	Volume decrease	8	10.63		8	10.75	
1 h Injection site clearance ratio	Volume increase	9	9.56	N-S	9	9.78	N-S
	Volume decrease	8	8.38		8	8.13	
2 h LN uptake ratio	Volume increase	9	8.11	N-S	9	7.67	N-S
	Volume decrease	8	10.00		8	10.50	
2 h Extremity uptake ratio	Volume increase	9	7.33	N-S	9	6.44	0.027*
	Volume decrease	8	10.88		8	11.88	
2 h Injection site clearance ratio	Volume increase	9	8.22	N-S	9	8.00	N-S
	Volume decrease	8	9.88		8	10.13	

^†^Volume difference ratio below zero was considered as volume increase

^§^Volume difference ratio above zero was considered as volume decrease

*Statistically significant; N-S, not statistically significant

Table 3 also shows the relationships between quantitative indicators and treatment response. There was no statistical significance in relation to early response, but 2-h EUR was statistically significant for late response (Fig. 3). Patients with a high 2-h EUR had a higher rate of volume reduction than did patients with a low 2-h EUR (*p* = 0.027).

The trend of dichotomous response in the 3 dermal backflow groups was evaluated, and there was a statistical significance in both early and late responses (Table 4). The proportion of postoperative volume reduction increased from the group with absent dermal backflow to the group with whole lower limb dermal backflow (early response, *p* = 0.040; late response, *p* = 0.040). Overall, there was no significant difference in the lymphoscintigraphic indicators predicting early and late dichotomous response.

**Table 4:** Treatment response according to dermal backflow pattern

	Dermal backflow pattern			
	Absent	Proximal only OR distal only	Whole lower limb (proximal and distal)	*p*-value
Early response (at 3 months)				
Volume increase^†^	4 (44.4%)	5 (55.6%)	0 (0.0%)	0.040*
Volume decrease^§^	1 (12.5%)	4 (50.0%)	3 (37.5%)	
Late response (at 1 year)				
Volume increase	4 (44.4%)	5 (55.6%)	0 (0.0%)	0.040*
Volume decrease	1 (12.5%)	4 (50.0%)	3 (37.5%)	

^†^Volume difference ratio below zero was considered as volume increase

^§^Volume difference ratio above zero was considered as volume decrease

*Statistically significant

## Discussion

This study aimed to investigate the factors predicting early and late treatment responses using lymphoscintigraphic indicators before LVA. The qualitative indicator analyses suggested that dermal backflow was a significant indicator, and that the surgical effect was better in the whole lower limb pattern or distal only pattern group. Patients with large amounts of dermal backflow or retention at the distal end would receive more help from bypass of the lymphatic-venous system. Several studies have been conducted on qualitative lymphoscintigraphic indicators and therapy response. J Yoo et al. reported that the severity of dermal backflow in secondary lower extremity lymphedema was a predictive factor for good therapeutic responders in multivariable analysis [18]. S Chiewvit et al. reported that dilated lymph vessels and dermal backflow were significantly associated with improved clinical results after LVA [20]. HO Kim et al. recently reported that the amount and pattern of dermal backflow were associated with treatment response in univariable and multivariable analyses [19], and this study is consistent with our findings. While several studies have used circumference difference [19, 27, 28], we used volume difference as a treatment response evaluation tool considering that this approach is suitable for detecting changes in edema.

The 2-h EUR was a significant factor in the quantitative indicator analyses. This corresponded to the results of qualitative analyses, as EUR is an indicator reflecting dermal backflow. Notably, 2-h EUR was more significant than 1-h EUR, suggesting that delayed imaging up to 2 h is helpful for lymphoscintigraphy. YB Kim et al. reported that extremity radioisotope uptake ratio was associated with early and late volume reduction in patients receiving complex decongestive therapy [29]. Our study shows that the same interpretation can apply to patients receiving LVA. Several studies have shown that quantitative indicators seen in lymphocytography are useful for diagnosis and prognosis [30]. A Szuba et al. reported a correlation between the axillary lymph node uptake index and the severity of lymphedema in patients with upper extremity lymphedema after breast cancer surgery [31]. JN Yoo et al. reported a significant correlation between quantitative lymphoscintigraphic factors and maximal circumferential difference in upper extremity lymphedema patients after breast cancer surgery [27]. Including the aforementioned study, several studies have used the ratio of quantitative indicators of edematous and non-edematous limbs [30, 32, 33]. Although this method is not available for bilateral lymphedema, it has the advantage of excellent reproducibility in several indicators [34]. There are also studies showing that the combination of qualitative and quantitative indicators augmented diagnostic efficacy [35, 36]. Our study demonstrated that combining qualitative and quantitative indicators was helpful not only in diagnostic but also prognostic fields.

The analysis performed by dividing dermal backflow into 3 groups according to amount and pattern showed a trend between the severity of edema and treatment response. As expected from the aforementioned analyses, as the amount of dermal backflow increased, the surgical effect was better. This finding is consistent with a previous study showing that patients with dermal backflow in both the thigh and calf are suitable for LVA [37]. Dermal backflow refers to a phenomenon in which lymphatic fluid leaks and accumulates in the skin and soft tissues by regurgitating the lymphatic flow because of increased pressure caused by occluded lymphatic vessels. Because LVA relieves these clogged lymphatic pathways, it can show a better therapeutic effect in patients with a large amount of dermal backflow. Therefore, checking the amount and site of dermal backflow as well as its presence in preoperative lymphoscintigraphy is important. These findings can be helpful to choose appropriate candidates for LVA who are expected to have a good postoperative response.

Since this study was conducted using radiopharmaceuticals, radiation issues could be involved. In general, the radiation exposure received by the patient due to lymphoscintigraphy is thought to be less than 1 mSv, which is higher than chest x-ray but similar to mammogram [38]. This is lower than the average allowable radiation dose of the general public (1 mSv/year), and much lower than commonly used imaging modalities such as bone scan and CT. Therefore, one shot of preoperative lymphoscintigraphy is unlikely to cause adverse effects in the patient. However, in the case of a young female patient or a patient who has already been exposed to a lot of radiation, even a small amount of exposure can be a burden, so a non-radiation technique such as MRI is recommended.

This study has some limitations. First, this study was performed with a small cohort of less than 30 patients, allowing this study as a preliminary study. In addition, the inhomogeneous patient composition including the etiology of lymphedema (primary and secondary) was another weak point. Second, the period of patient enrollment was relatively long. Although the surgical method or surgeon has not changed significantly within this decade, other factors might have an influence, such as advances in non-surgical methods. Third, volume difference ratio was used as an assessment tool in this study, but MRI is commonly used objective evaluation technique [39]. Routine MRI work-up was not performed in patients with lymphedema in our institution so the use of MRI is recommended in the future studies. Finally, multivariable analysis could not be performed due to the lack of significant results in univariable analyses for clinical indicators. Therefore, a further study with a larger number of subjects is warranted.

## Conclusions

Preoperative lymphoscintigraphy is useful to predict both early and late therapy response in patients with lower extremity lymphedema undergoing LVA. Both the dermal backflow pattern and extremity uptake ratio are predictive lymphoscintigraphic indicators. In other words, as the amount of dermal backflow increased, better postoperative therapeutic outcome may be expected. Although the lymphoscintigraphic indicators predicting early and late treatment responses were similar, the 2-h imaging indicators were more useful than the 1-h imaging indicators.

## Data Availability

The datasets used and/or analysed during the current study are available from the corresponding author on reasonable request.

## Abbreviations

LVA: Lymphovenous anastomosis
EUR: Extremity uptake ratio

## References

[1] Jensen MR, Simonsen L, Karlsmark T, Bulow J (2010). Lymphoedema of the lower extremities–background, pathophysiology and diagnostic considerations. Clin Physiol Funct Imaging.

[2] Grada AA, Phillips TJ (2017). Lymphedema: Pathophysiology and clinical manifestations. J Am Acad Dermatol.

[3] Paim CR, de Paula Lima ED, Fu MR, de Paula LA, Cassali GD (2008). Post lymphadenectomy complications and quality of life among breast cancer patients in Brazil. Cancer Nurs.

[4] Torgbenu E, Luckett T, Buhagiar MA, Chang S, Phillips JL (2020). Prevalence and incidence of cancer related lymphedema in low and middle-income countries: a systematic review and meta-analysis. BMC Cancer.

[5] Beesley VL, Rowlands IJ, Hayes SC, Janda M, O'Rourke P, Marquart L (2015). Incidence, risk factors and estimates of a woman's risk of developing secondary lower limb lymphedema and lymphedema-specific supportive care needs in women treated for endometrial cancer. Gynecol Oncol.

[6] Gallagher K, Marulanda K, Gray S (2018). Surgical intervention for lymphedema. Surg Oncol Clin N Am.

[7] Chang DW, Masia J, Garza R, Skoracki R, Neligan PC (2016). Lymphedema: surgical and medical therapy. Plast Reconstr Surg.

[8] Yoshida S, Koshima I, Sasaki A, Fujioka Y, Nagamatsu S, Yokota K, et al. Mechanical Dilation Using Nylon Monofila ment Aids Multisite Lymphaticovenous Anastomosis Through Improving the Quality of Anastomosis. Ann Plast Sur g. 2019;82(2):201–6. 10.1097/SAP.0000000000001681 30557189

[9] Yoshida S, Koshima I, Sasaki A, Fujioka Y, Nagamatsu S, Yokota K, et al. Mechanical Dilation With a Nylon Monofil ament for 0.1-mm Anastomoses. Ann Plast Surg. 2019;82(2):233-6. 10.1097/SAP.0000000000001632 30300221

[10] Yoshida S, Koshima I, Imai H, Eldahshoury TEM, Sasaki A, Fujioka Y, et al. Line production system for multiple lym phaticovenular anastomoses. J Plast Reconstr Aesthet Surg. 2019;72(8):1334-9. 10.1016/j.bjps.2019.03.038 31056432

[11] Yoshida S, Koshima I, Imai H, Sasaki A, Nagamatsu S, Yokota K. Lymphaticovenular anastomosis for recurrent cellu litis in a dementia patient with lymphedema. J Vasc Surg Cases Innov Tech. 2020;6(3):340-3. 10.1016/j.jvscit.2020.06.007 PMC7371726 32715167

[12] Yoshida S, Koshima I, Hamada Y, Sasaki A, Fujioka Y, Nagamatsu S, et al. Lymphovenous Anastomosis Aids Woun d Healing in Lymphedema: Relationship Between Lymphedema and Delayed Wound Healing from a View of Imm une Mechanisms. Adv Wound Care (New Rochelle). 2019;8(6):263-9. 10.1089/wound.2018.0871 PMC6906758 31832276

[13] Kim YH, Choi JY, Kim YW, Kim DI, Do YS, Hwang JH (2009). Characterization of congenital vascular malformation in the extremities using whole body blood pool scintigraphy and lymphscintigraphy. Lymphology.

[14] Williams WH, Witte CL, Witte MH, McNeill GC (2000). Radionuclide lymphangioscintigraphy in the evaluation of peripheral lymphedema. Clin Nucl Med.

[15] Pecking AP, Albérini JL, Wartski M, Edeline V, Cluzan RV (2008). Relationship between lymphoscintigraphy and clinical findings in lower limb lymphedema (LO): toward a comprehensive staging. Lymphology.

[16] Hwang JH, Kwon JY, Lee KW, Choi JY, Kim BT, Lee BB (1999). Changes in lymphatic function after complex physical therapy for lymphedema. Lymphology.

[17] Hwang JH, Choi JY, Lee JY, Hyun SH, Choi Y, Choe YS (2007). Lymphscintigraphy predicts response to complex physical therapy in patients with early stage extremity lymphedema. Lymphology.

[18] Yoo J, Choi JY, Hwang JH, Kim DI, Kim YW, Choe YS (2014). Prognostic value of lymphoscintigraphy in patients with gynecological cancer-related lymphedema. J Surg Oncol.

[19] Kim HO, Woo KJ, Kim BS, Kang SY, Moon BS, Yoon HJ (2021). Lymphoscintigraphic findings as indicators of lymphaticovenous anastomosis outcome in patients with extremity lymphedema: a retrospective cohort study. Clin Nucl Med.

[20] Chiewvit S, Kumnerdnakta S (2017). Lymphoscintigraphic findings that predict favorable outcome after lymphaticovenous anastomosis. Lymphology.

[21] Vaqueiro M, Glovicski P, Fisher J, Hollier LH, Schirger A, Wahner HW (1986). Lymphoscintigraphy in lymphedema: an aid to microsurgery. J Nucl Med.

[22] Kung TA, Champaneria MC, Maki JH, Neligan PC (2017). Current concepts in the surgical management of lymphedema. Plast Reconstr Surg.

[23] Seo Y-D, Jeong J-H, Moon J-W, Yun S-H, Kim Y-S, Kang S-H (2009). Performance evaluation of substitution radiopharmaceutical according to restriction of the radiocolloids in lymphoscintigraphy. Korean J Nucl Med Technol.

[24] Szuba A, Rockson SG (1998). Lymphedema: classification, diagnosis and therapy. Vasc Med.

[25] de Carvalho RM, Perez MdCJ, Miranda F, Jr. Assessment of the intraobserver and interobserver reliability of a communicating vessels volumeter to measure wrist-hand volume. Phys Ther. 2012;92(10):1329–37. 10.2522/ptj.20110169 22677294

[26] Al-Niaimi F, Cox N (2009). Cellulitis and lymphoedema: a vicious cycle. J Lymphoedema.

[27] Yoo JN, Cheong YS, Min YS, Lee SW, Park HY, Jung TD (2015). Validity of quantitative lymphoscintigraphy as a lymphedema assessment tool for patients with breast cancer. Ann Rehabil Med.

[28] Ho OA, Chu S-Y, Huang Y-L, Chen W-H, Lin C-Y, Cheng M-H. Effectiveness of vascularized lymph node transfer for extremity lymphedema using volumetric and circumferential differences. Plast Reconstr Surg Glob Open. 2019;7(2). 10.1097/GOX.0000000000002003 PMC6416128 30881819

[29] Kim YB, Hwang JH, Kim TW, Chang HJ, Lee SG (2012). Would complex decongestive therapy reveal long term effect and lymphoscintigraphy predict the outcome of lower-limb lymphedema related to gynecologic cancer treatment?. Gynecol Oncol.

[30] Kim P, Lee JK, Lim OK, Park HK, Park KD (2017). Quantitative lymphoscintigraphy to predict the possibility of lymphedema development after breast cancer surgery: retrospective clinical study. Ann Rehabil Med.

[31] Szuba A, Pyszel A, Jedrzejuk D, Janczak D, Andrzejak R (2007). Presence of functional axillary lymph nodes and lymph drainage within arms in women with and without breast cancer-related lymphedema. Lymphology.

[32] Kim K, Kim IJ, Pak K, Kim SJ, Choi SJ, Park H (2020). The feasibility of quantitative parameters of lymphoscintigraphy without significant dermal backflow for the evaluation of lymphedema in post-operative patients with breast cancer. Eur J Nucl Med Mol Imaging.

[33] Szuba A, Strauss W, Sirsikar S, Rockson S (2002). Quantitative radionuclide lymphoscintigraphy predicts outcome of manual lymphatic therapy in breast cancer-related lymphedema of the upper extremity. Nucl Med Commun.

[34] Devoogdt N, Van den Wyngaert T, Bourgeois P, Lambrechts M, Van Kampen M, De Groef A (2014). Reproducibility of lymphoscintigraphic evaluation of the upper limb. Lymphat Res Biol.

[35] Dabrowski J, Merkert R, Kuśmierek J (2008). Optimized lymphoscintigraphy and diagnostics of lymphatic oedema of the lower extremities. Nucl Med Rev Cent East Eur.

[36] Weissleder H, Weissleder R (1988). Lymphedema: evaluation of qualitative and quantitative lymphoscintigraphy in 238 patients. Radiology.

[37] Maegawa J, Mikami T, Yamamoto Y, Satake T, Kobayashi S (2010). Types of lymphoscintigraphy and indications for lymphaticovenous anastomosis. Microsurgery.

[38] Giammarile F, Alazraki N, Aarsvold JN, Audisio RA, Glass E, Grant SF (2013). The EANM and SNMMI practice guideline for lymphoscintigraphy and sentinel node localization in breast cancer. Eur J Nucl Med Mol Imaging.

[39] Cellina M, Gibelli D, Floridi C, Oliva G (2020). Volumetric analysis of non-contrast magnetic resonance lymphangiography in patients affected by lower extremities primary lymphedema. Radiol Med.

